# Species of the genus *Thressa* Walker, 1860 from China (Diptera, Chloropidae)

**DOI:** 10.3897/zookeys.129.1144

**Published:** 2011-09-16

**Authors:** Xiao-Yan Liu, Ding Yang, Emilia P. Nartshuk

**Affiliations:** 1 Department of Entomology, China Agricultural University, Beijing 100193, China; 2 Zoological Institute, Russian Academy of Sciences, St. Petersburg 199034, Russia

**Keywords:** Diptera, Chloropidae, *Thressa*, new species, China

## Abstract

A review of the species of the genus *Thressa* from China is provided. The following four species are described as new to science: *Thressa bimaculata* **sp. n.**, *Thressa daiyunshana* **sp. n.**, *Thressa foliacea* **sp. n.** and *Thressa longimaculata* **sp. n.** One species, *Thressa flavior* (Duda), is recorded from China for the first time. A key to the species of the *Thressa* from China is given.

## Introduction

The genus *Thressa* was erected by Walker (1860). It belongs to the *Thaumatomyia* genus group (Andersson 1977), with the following characters: body (Figs 1, 2) small in size, black species; head higher than long, very large and broad; gena linear; parafacial not visible; ocellar triangle large, covering most of frons, smooth, black with metallic luster; antenna brownish, more or less darkened, scape and pedicel short, postpedicel oval, about twice or more as long as broad, distinctly pubescent; arista peculiar in form, pectinate without marginal pubescence; scutum prominently convex; scutellum short, rounded, basally flat (Andersson 1977; Kanmiya 1983 ). There are 16 species known worldwide, of which twelve species are only known from the Oriental Region (Sabrosky 1977; Kanmiya 1983; Yang 1992; Nartshuk 1993), three species from the Australian Region (which are also distributed in the Oriental Region) (Sabrosky 1977; Spencer 1986), and one species, *Thressa spuria* (Thomson), from the Palaearctic Region (China and Pakistan) (Sabrosky 1977; Kanmiya 1983). Nartshuk (1993) reviewed world species, and gave a key to world species except for two species described by Yang (1992) not included.

**Figure:**
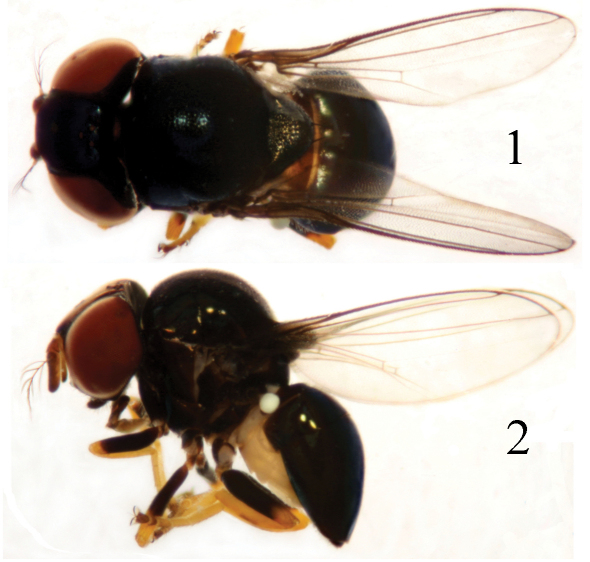
**Figures 1–2.** *Thressa guizhouensis* Yang, male. **1** body, dorsal view **2** body, lateral view.

Presently, five species are known to occur in China (Thomson 1869; Becker and de Meijere 1913; Becker 1916; Yang 1992), including two species from Taiwan, *Thressa beckeri* (de Meijere) and *Thressa cyanescens* (Becker), and three species from continental China, *Thressa spuria* (Thomson), *Thressa guizhouensis* Yang and *Thressa maculata* Yang. Recently, we studied *Thressa* collections including types of two species described by Yang (1992) from the Entomological Museum of China Agricultural Univeristy, and found four new species and one species newly recorded from China, which are described in the present paper. A key to the species of the genus from China is presented.

## Material and methods

Specimens were studied and illustrated with a ZEISS Stemi 2000–c. Genitalic preparations were made by macerating the apical portion of the abdomen in warm 10% NaOH for 17–20 min, after examination it was transferred to fresh glycerine and stored in a microvial pinned below the specimen. Specimens are deposited in the Entomological Museum of China Agricultural University (CAU), Beijing.

The following abbreviations are used:

ap sc apical scutellar seta

oc ocellar seta

orb orbital seta

if interfrontal seta

vti medial vertical seta

vte lateral vertical seta

pvt postvertical seta.

## Taxonomy

### A key to the species of *Thressa* from China (modified from Nartshuk 1993)

**Table:** 

1	Wing hyaline, with a brown spot near wing apex	2
–	Wing hyaline, without a brown spot near wing apex	6
2	Costal cell and marginal cell somewhat tinged with brown	*Thressa longimaculata* sp. n.
–	Only costal cell somewhat tinged with brown	3
3	Abdomen entirely black, shiny	4
–	Abdomen not entirely black, distal portion with yellow in varying degrees	5
4	Legs yellow, but femora black centrally in female; surstylus basally broad, distally narrowed	*Thressa beckeri* (de Meijere, 1913)
–	Legs black, but distal part of femora, entire tibiae and tarsi yellow; surstylus short, leaf-shaped	*Thressa foliacea* sp. n.
5	Legs yellow, except for basal fore coxae, mid coxae black; pregonite about as long as postgonite	*Thressa daiyunshana* sp. n.
–	Legs yellow, except for fore and mid coxae black, fore femora blackish, only distal ends yellow; pregonite shorter than postgonite	*Thressa maculata* Yang, 1992
6	Scutum with yellow spot anterior to scutellum	7
–	Scutum entirely black, without yellow spot anterior to scutellum	8
7	Scutum with 2 slightly impressed short and shallow depressions on dorsocentral lines, and 2 small yellow spots on both sides of scutum anterior to scutellum	*Thressa bimaculata* sp. n.
–	Scutum without impressed short and shallow depressions on dorsocentral lines, with minutely granulated surface, and a yellow transverse stripe anterior to scutellum	*Thressa flavior* (Duda, 1934)
8	Epandrium with a pair of band-like sclerites along posterodorsal notch; cercus with a concavity on ventral margin	*Thressa cyanescens* (Becker, 1916)
–	Epandrium without a pair of band-like sclerites along posterodorsal notch; cercus without a concavity on ventral margin	9
9	Frons slightly longer than wide (33:28); costal cell almost colorless	*Thressa spuria* (Thomson, 1869)
–	Frons about as long as wide; costal cell somewhat tinged with brown	*Thressa guizhouensis* Yang, 1992

### Thressa bimaculata sp. n.

urn:lsid:zoobank.org:act:BE223EEF-761E-409D-B4B6-88F7EA931958

http://species-id.net/wiki/Thressa_bimaculata

Figs 3 11 

#### Diagnosis.

 Scutum with 2 small yellow spots on both sides anterior to scutellum. Dorsal portion of katepisternum with a small band-like yellow spot. Legs yellow except tarsomere 5 brown. Abdomen black except tergite 5 medially black and laterally yellow.

#### Description.

 Male. Body length 2.0 mm, wing length1.8 mm.

Head (Figs 3, 4) black without microtomentum, about 0.75 times as long as high, wider than thorax; face sometimes concave in lateral view, bright brown; epistoma yellow; frons black, about as long as wide, projecting only slightly in front of eye, almost entirely occupied by broad ocellar triangle; gena linear; vibrissal angle obtuse; parafacial black, narrow; postgena black; clypeus light black. Ocellar triangle very large and broad, smooth, black, shiny, reaching to anterior margin of frons with broad apex; ocellar tubercle black. Occiput black. Cephalic setae and setulae black, weakly developed; *if* extremely short, in 1 row on the surface of the triangle; *orb* very minute, upright; *oc* extremely small; *pvt* small hair-like, upright, convergent; *vte* shorter than *pvt* and *vti* indistinct. Antenna dark brown, with thick grayish microtomentum, but postpedicel missing in holotype. Proboscis and palpus black with blackish setulae.

Thorax (Figs 5, 6) shiny black without microtomentum, evenly covered with short setulae. Scutum strongly convex, almost as long as wide, with 2 slightly impressed short and shallow depressions on dorsocentral lines, and 2 small yellow spots on both sides of scutum anterior to scutellum. Thoracic pleuron darkish brown except for katepimeron and anepimeron with some pale gray microtomentum; dorsal portion of katepisternum with a small band-like yellow spot. Scutellum about 0.7 times as long as wide; *ap sc* short, distinctly shorter than scutellum. Setae and setulae on thorax black. Legs yellow except tarsomere 5 brown. Setulae on legs yellow, but apical portion of tarsi with some brown setulae. Tibial organ distinct, oblong. Wing (Fig. 7) about 2.7 times as long as wide, hyaline without brown spot near wing apex, costal cell somewhat tinged with brown; veins brown. Relative lengths of 2nd : 3rd : 4th costal sections = 5 : 3 : 1; discal cell narrow and long; crossveins r-m and m-m not approximate, r-m at basal 1/3 of discal cell. Halter pale yellow on knob, brown on stem.

**Figure:**
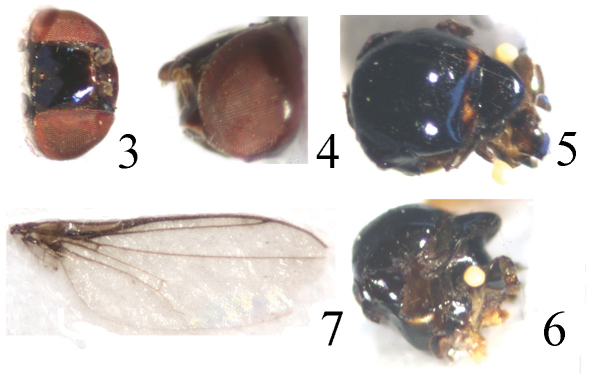
**Figures 3–7.** *Thressa bimaculata* sp. n., male. **3** head, dorsal view **4** head, lateral view **5** mesonotum and scutellum, dorsal view **6** mesonotum and scutellum, lateral view **7** wing.

Abdomen shiny black except for tergite 5 medially black and laterally yellow; venter yellow. Setulae on abdomen black. Male genitalia (Figs 8–11): Epandrium short tubular, weakly sclerotized, yellow with yellow long setulae, with 2 band-like sclerites along posterodorsal notch; surstylus basally broad with short spinous setulae, distally narrowed, attached to epandrium anteroventrally. Cercus small, semicircular in dorsal view. Gonites arranged in a row; postgonite slightly incurved distally with pointed apex, with some sensory setulae, basally gradually narrowed; pregonite shorter than postgonite, basally broad and round, distally narrowed; basiphallus longer than wide, cylindrical; distiphallus cylindrical, longer than basiphallus, extended to basal 1/4 of pregonite, largely membranous but weakly sclerotized on apical end; phallapodeme long, extended near base of basiphallus, with basal stalk broad in lateral view. Hypandrium narrow.

Female. Unknown.

**Figure:**
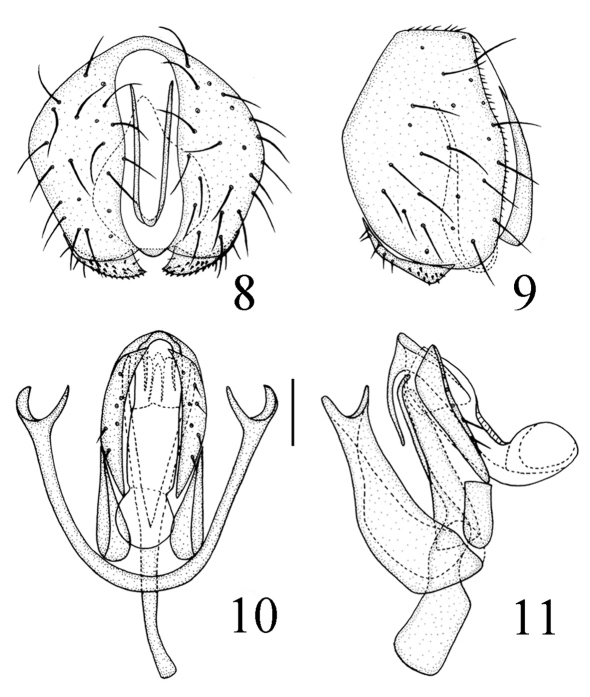
**Figures 8–11.** *Thressa bimaculata* sp. n., male. **8** epandrium, posterior view **9** epandrium, lateral view **10** hypandrium and phallic complex, ventral view **11** hypandrium and phallic complex, lateral view. Scale bar = 0.05mm.

#### Type material.

Holotype ♂, Yunnan: Mengla (21°29'N, 101°33'E, 780m), 9. V. 2009, Guoquan Wang. Male type specimen dry mounted on label laterally on right thorax; postpedicel is missing.

#### Distribution.

 China: Yunnan.

#### Remarks.

The new species is somewhat similar to *Thressa cyanescens* (Becker) in the scutum with the slightly impressed short and shallow depressions on dorsocentral lines and the abdomen shiny black except tergite 5 medially black and laterally yellow. But it can be separated from the latter by the scutum with 2 small yellow spots on both sides anterior to the scutellum; the dorsal portion of the katepisternum with one small band-like yellow spot; the cercus semicircular in dorsal view. In *Thressa cyanescens*, the scutum is entirely shiny black; the thoracic pleuron is entirely shiny black except for the microtomentose katepimeron; the cercus has a concavity on the ventral margin (Becker 1916; Kanmiya 1983; Nartshuk 1993).

#### Etymology.

The specific name is from the Latin *bi-* (“two”) and *maculatus* (“maculate”), refers to the scutum with 2 small yellow spots.

### Thressa daiyunshana sp. n.

urn:lsid:zoobank.org:act:5165C077-244C-4F30-8507-BD41FA133F72

http://species-id.net/wiki/Thressa_daiyunshana

Figs 12 19 

#### Diagnosis.

Thoracic pleuron blackish brown except for posterodorsal portion of katepisternum with a triangular yellowish brown spot; anterodorsal portion of katepimeron with a triangular yellow spot. Cercus triangular in ventral view. Pregonite about as long as postgonite.

#### Description.

 Male. Body length2.9 mm, wing length2.2 mm.

Head (Fig. 12) black without microtomentum, about 0.8 times as long as high, wider than thorax; face sometimes concave in lateral view, bright brown with a yellow medial stripe more or less on dorsal 1/2; epistoma yellow; frons black, about as long as wide, projecting only slightly in front of eye, almost entirely occupied by broad ocellar triangle; gena narrowed, about 1/10 times as high as postpedicel; vibrissal angle obtuse; parafacial black, linear; postgena black; clypeus light black. Ocellar triangle very large and broad, smooth, black, shiny metallic blue, reaching to anterior margin of frons with broad apex; ocellar tubercle black. Occiput black. Cephalic setae and setulae black, weakly developed; *if* extremely short, in 1 row on the surface of the triangle; *orb* very minute, upright; *oc* subequal to *orb*; *pvt* small hair-like, upright, convergent; *vte* shorter than *pvt* and *vti* indistinct. Antenna darkish brown with thick grayish microtomentum, but pedicel with yellow ventral surface, postpedicel yellow at basoventral portion; postpedicel 2 times as long as wide, parallel-sided; arista pectinate, with 5 branches dorsally and 4 ventrally, apically with some short setulae, black except for basal segment yellow. Proboscis yellow with yellowish setulae and palpus black with black setulae.

Thorax (Figs 13, 14) shiny black without microtomentum, with granulated microsculpture, evenly covered with short setulae. Scutum strongly convex, almost as long as wide. Thoracic pleuron blackish brown except for posterodorsal portion of katepisternum with a triangular yellowish brown spot; anterodorsal portion of katepimeron with a triangular yellow spot. Scutellum about 0.5 times as long as wide; *ap sc* short, distinctly shorter than scutellum. Setae and setulae on thorax black. Legs yellow except for basal fore coxae, mid coxae, tarsomeres 4–5 black. Setulae on legs yellow, but apical portion of tarsi with some brown setulae. Tibial organ distinct, oblong. Wing (Fig. 15) about 2.9 times as long as wide, hyaline with a brown spot near wing apex, costal cell somewhat tinged with brown; veins brown. Relative lengths of 2nd : 3rd : 4th costal sections = 5 : 2.5 : 1; discal cell narrow and long; crossveins r-m and m-m not approximate, r-m at basal 1/3 of discal cell. Halter pale yellow on knob, brown on stem.

**Figure:**
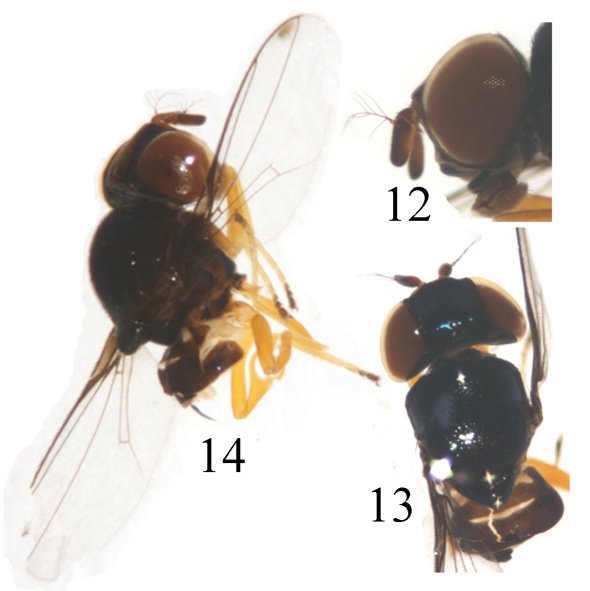
**Figures 12–14.** *Thressa daiyunshana* sp. n., male. **12** head, lateral view **13** head and thorax, dorsal view **14** head and thorax, lateral view.

Abdomen shiny black except for distal 1/3 of tergite 3 and tergite 4 medially yellow, tergite 5 entirely yellow; venter yellow. Setulae on abdomen brown. Male genitalia (Figs 16–19): Epandrium short tubular, weakly sclerotized, yellow with long yellow setulae; surstylus basally broad with short spinous setulae, distally narrowed, attached to epandrium anteroventrally. Cercus triangular in ventral view. Gonites arranged in a row; postgonite slightly incurved distally with pointed apex, basally gradually narrowed, with some sensory setulae; pregonite about as long as postgonite, basally broad and round, distally narrowed; basiphallus longer than wide, cylindrical; distiphallus cylindrical, longer than basiphallus, beyond lower margin of hypandrium, largely membranous but weakly sclerotized on apex; phallapodeme long, extended near base of basiphallus, with basal stalk broad in lateral view. Hypandrium narrow.

Female. Unknown.

**Figure:**
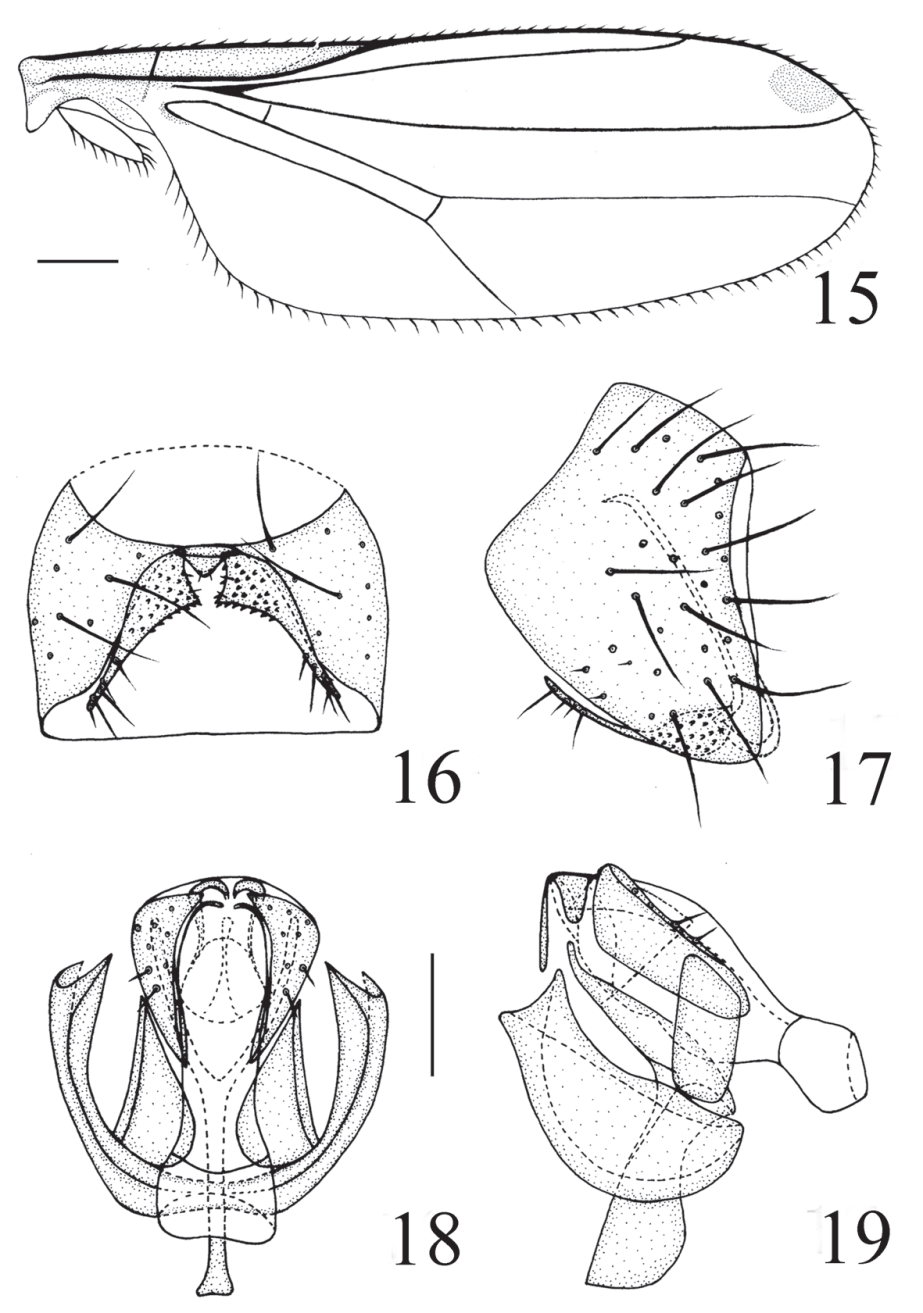
**Figures 15–19.** *Thressa daiyunshana* sp. n., male. **15** wing **16** epandrium, ventral view **17** epandrium, lateral view **18** hypandrium and phallic complex, ventral view **19** hypandrium and phallic complex, lateral view. Scale bar = 0.2mm (15), Scale bar = 0.05mm (16–19).

#### Type material.

Holotype ♂, Fujian: Dehua, Daiyunshan (25°47'N, 118°15'E, 720m), 11. VII. 2010, Xiaoyan Liu. Male type specimen was stored in 75% ethanol.

#### Distribution.

 China: Fujian.

#### Remarks.

 The new species is somewhat similar to *Thressa apicalis* (de Meijere) in the wing with a brown spot near the wing apex and the abdomen largely yellow. But it can be separated from the latter by the posterodorsal portion of the katepisternum with a triangular yellowish brown spot; the anterodorsal portion of the katepimeron with a triangular yellow spot; the legs yellow except for the basal fore coxae, mid coxae black. In *Thressa apicalis*, the dorsal portion of the katepisternum has one elongated yellow spot; the legs are yellow except the basal fore coxae black (de Meijere 1910; Nartshuk 1993).

#### Etymology.

The species isnamed after the type locality Daiyunshan.

### Thressa foliacea sp. n.

urn:lsid:zoobank.org:act:692F9739-E69F-414B-9A14-307B16AA53B2

http://species-id.net/wiki/Thressa_foliacea

Figs 20 28 

#### Diagnosis.

Antenna black; postpedicel 2.5 times as long as wide. Thoracic pleuron bright black except katepisternum, anepimeron and katepimeron with some pale gray microtomentum; paratergite and dorsal portion of anepisternum with a trapezoidal, bright yellow spot. Surstylus short, leaf-shaped.

#### Description.

 Male.Body length2.3 mm, wing length1.9 mm.

Head (Figs 20, 21) black without microtomentum, about 0.9 times as long as high, wider than thorax; face sometimes concave in lateral view, bright brown with two yellow spots below antenna; epistoma yellow; frons black, 1.1 times as long as wide, projecting only slightly in front of eye, almost entirely occupied by broad ocellar triangle; gena narrow; vibrissal angle obtuse; parafacial black, linear; postgena black; clypeus light black. Ocellar triangle very large and broad, smooth, black, shiny metallic blue, reaching to anterior margin of frons with broad apex; ocellar tubercle black. Occiput black. Cephalic setae and setulae black, weakly developed; *if* extremely short, in 1 row on the surface of the triangle; *orb* very minute, upright; *oc* subequal to *orb*; *pvt* small hair-like, upright, convergent; *vte* shorter than *pvt* and *vti* indistinct. Antenna black with thick grayish microtomentum; postpedicel 2.5 times as long as wide, parallel-sided; arista missing in holotype. Proboscis and palpus blackish brown with brownish setulae.

Thorax (Figs 22, 23) shiny black without microtomentum, with granulated microsculpture, evenly covered with short setulae. Scutum strongly convex, almost as long as wide. Thoracic pleuron bright black except for katepisternum, anepimeron and katepimeron with some pale gray microtomentum; paratergite and dorsal portion of anepisternum with a trapezoidal, bright yellow spot. Scutellum about 0.5 times as long as wide; *ap sc* short, distinctly shorter than scutellum. Setae and setulae on thorax black. Legs black except for distal part of femora, entire tibiae and tarsi yellow. Setulae on legs yellow, but apical portion of tarsi with some brown setulae. Tibial organ distinct, oblong. Wing (Fig. 24) about 2.9 times as long as wide, hyaline with a brown spot near wing apex, costal cell somewhat tinged with brown; veins brown. Relative lengths of 2nd : 3rd : 4th costal sections = 5 : 2.5 : 1; discal cell narrow and long; crossveins r-m and m-m not approximate, r-m at basal 1/3 of discal cell. Halter pale yellow on knob, brown on stem.

**Figure:**
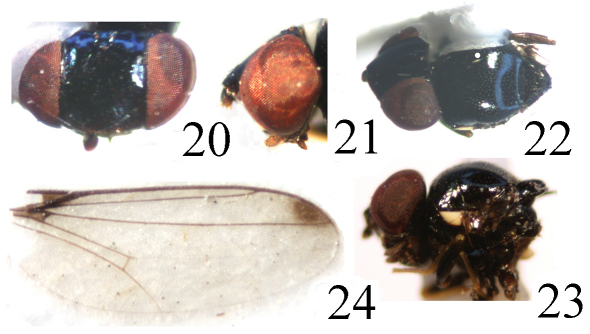
**Figures 20–24.** *Thressa foliacea* sp. n., male. **20** head, dorsal view **21** head, lateral view **22** mesonotum and scutellum, dorsal view **23** mesonotum and scutellum, lateral view **24** wing.

Abdomen shiny black; venter yellow. Setulae on abdomen black. Male genitalia (Figs 25–28): Epandrium short tubular, weakly sclerotized, black with long black setulae; surstylus short, leaf-shaped with short spinous setulae. Cercus with a concavity on ventral margin. Gonites arranged in a row; postgonite slightly incurved distally with blunt apex, basal 1/4 obviously narrowed, with some sensory setulae; pregonite shorter, about 0.5 times as long as postgonite, basally broad, distally narrowed; basiphallus longer than wide, cylindrical; distiphallus cylindrical, longer than basiphallus, beyond lower margin of hypandrium, largely membranous but weakly sclerotized on apical end; phallapodeme long, extended near base of basiphallus, with basal stalk short and broad in lateral view. Hypandrium broad.

Female. Unknown.

**Figure:**
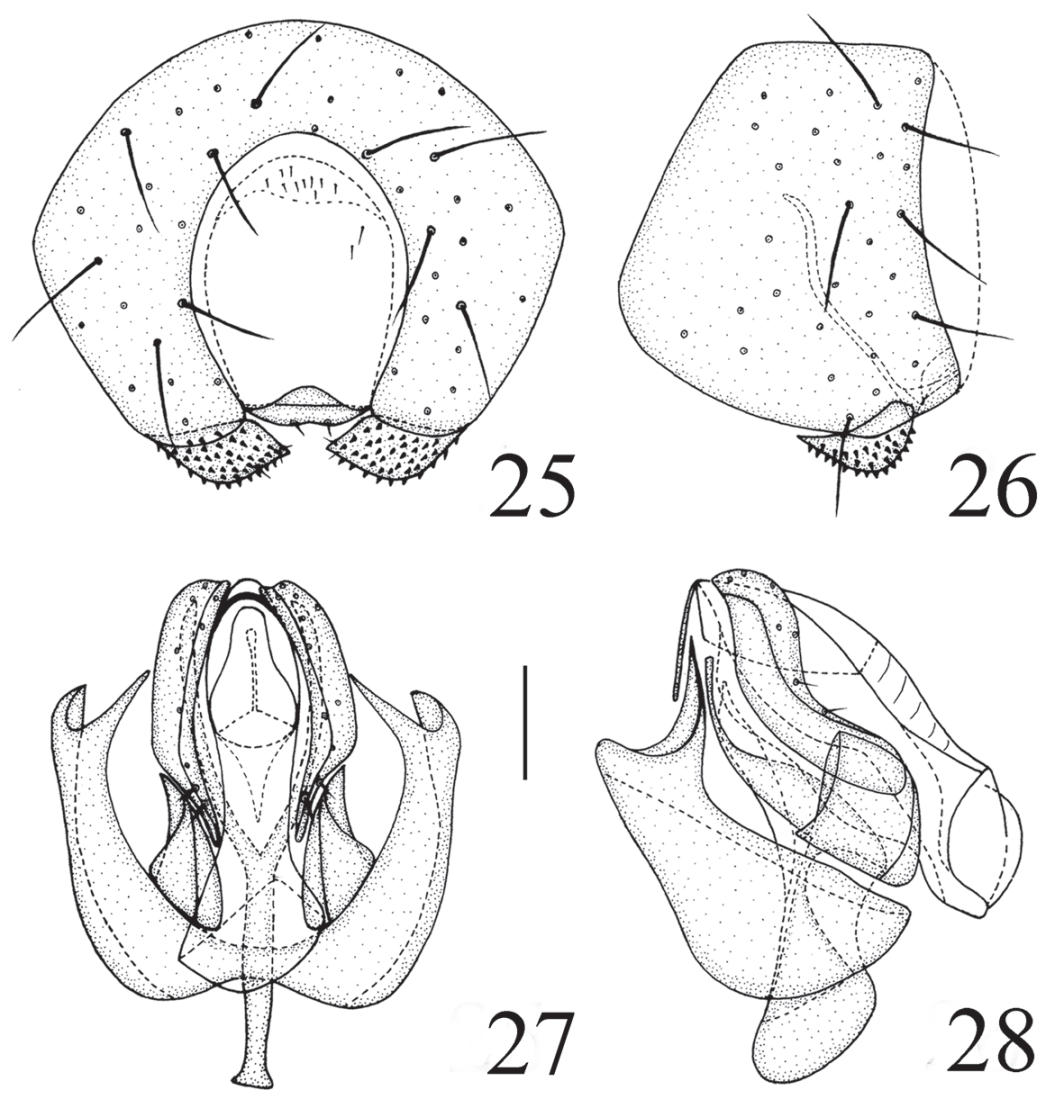
**Figures 25–28.** *Thressa foliacea* sp. n., male. **25** epandrium, posterior view **26** epandrium, lateral view **27** hypandrium and phallic complex, ventral view **28** hypandrium and phallic complex, lateral view. Scale bar = 0.05mm.

#### Type material.

Holotype ♂, Hainan: Baisha (19°11'N, 109°25'E, 430m), 19. X. 2007, Xingyue Liu. Male type specimen dry mounted on label laterally on right thorax; arista is missing.

#### Distribution.

 China: Hainan.

#### Remarks. 

The new species is somewhat similar to *Thressa punctifera* (de Meijere) in the wing with a brown spot near the wing apex and the abdomen shiny black. But it can be separated from the latter by the paratergite and dorsal portion of the anepisternum with one trapezoidal, bright yellow spot; the legs black except for the distal part of femora, entire tibiae and tarsi yellow; the surstylus short, leaf-shaped. In *Thressa punctifera*, the katepisternum has one narrow yellow spot; the legs are yellow except for the fore femora brownish; the surstylus is broad basally and narrow distally (de Meijere 1910; Nartshuk 1993).

#### Etymology. 

The specific name is from the Latin *foliaceus* (“leaf-shaped”), refers to the leaf-shaped surstylus.

### Thressa longimaculata sp. n.

urn:lsid:zoobank.org:act:39D8C4B4-1E6F-486F-BE05-BAD2A3B96F40

http://species-id.net/wiki/Thressa_longimaculata

Figs 29 36 

#### Diagnosis.

Thoracic pleuron brown except for katepisternum and katepimeron with black lower portion; paratergite and posterodorsal portion of anepisternum with a triangular yellow spot. Wing hyaline with a long brown spot near wing apex, costal cell and marginal cell somewhat tinged with brown. Cercus semicircular in dorsal view. Postgonite with basal 1/3 obviously narrowed.

#### Description.

 Male. Body length 2.6 mm, wing length 2.0 mm

Head (Fig. 29) black without microtomentum, about 0.85 times as long as high, wider than thorax; face sometimes concave in lateral view, bright brown with a yellow medial stripe more or less on dorsal 1/2; epistoma yellow; frons black, about as long as wide, projecting only slightly in front of eye, almost entirely occupied by broad ocellar triangle; gena narrow, about 1/10 times as high as postpedicel; vibrissal angle obtuse; parafacial black, linear; postgena black; clypeus light black. Ocellar triangle very large and broad, smooth, black, shiny metallic blue, reaching to anterior margin of frons with broad apex; ocellar tubercle black. Occiput black. Cephalic setae and setulae black, weakly developed; *if* extremely short, in 1 row on the surface of the triangle; *orb* very minute, upright; *oc* extremely small; *pvt* small hair-like, upright, convergent; *vte* short than *pvt* and *vti* indistinct. Antenna darkish brown with thick grayish microtomentum, but pedicel with yellow ventral surface, postpedicel yellow at basoventral portion; postpedicel 2 times as long as wide, parallel-sided; arista pectinate, with 5 branches dorsally and 4 ventrally, apically with some short setulae, black except for basal segment yellow. Proboscis yellow with yellowish setulae and palpus black with black setulae.

Thorax (Figs 30, 31) shiny black without microtomentum, with granulated microsculpture, evenly covered with short setulae. Scutum strongly convex, almost as long as wide. Thoracic pleuron brown except for katepisternum and katepimeron with black lower portion; paratergite and posterodorsal portion of anepisternum with a triangular yellow spot. Scutellum about 0.5 times as long as wide; *ap sc* short, distinctly shorter than scutellum. Setae and setulae on thorax black. Legs yellow except for basal portion of mid coxae, tarsomere 5 brown. Setulae on legs yellow, but apical portion of tarsi with some brown setulae. Tibial organ distinct, oblong. Wing (Fig. 32) about 3.1 times as long as wide, hyaline with a brown spot near wing apex, costal cell and marginal cell somewhat tinged with brown; veins brown. Relative lengths of 2nd : 3rd : 4th costal sections = 5 : 2.5 : 1; discal cell narrow and long; crossveins r-m and m-m not approximate, r-m at basal 1/3 of discal cell. Halter pale yellow on knob, brown on stem.

**Figure:**
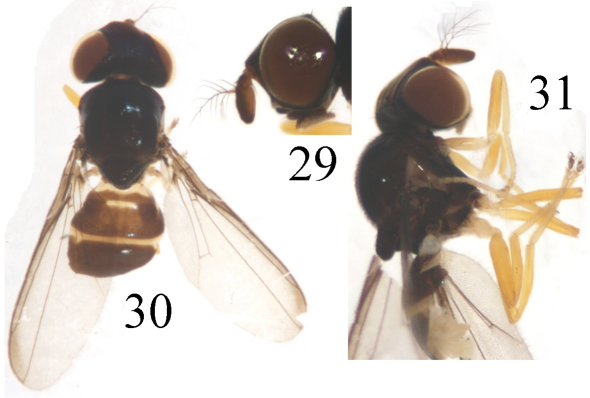
**Figures 29–31.** *Thressa longimaculata* sp. n., male. **29** head, lateral view **30** head and thorax, dorsal view **31** head and thorax, lateral view.

Abdomen shiny blackish brown except tergite 5 yellow with a M-shaped black spot; venter yellow. Setulae on abdomen brown. Male genitalia (Figs 33–36): Epandrium short tubular, weakly sclerotized, yellow with long yellow setulae; surstylus basally broad with short spinous setulae, distally narrowed, attaching to epandrium anteroventrally. Cercus semicircular in dorsal view. Gonites arranged in a row; postgonite gradually narrowed distad and slightly convergent, basal 1/3 obviously narrowed, with sensory setulae; pregonite shorter than postgonite, basally broad and round, distally narrowed; basiphallus longer than wide, cylindrical; distiphallus cylindrical, longer than basiphallus, beyond lower margin of hypandrium, largely membranous but weakly sclerotized on apical end; phallapodeme long, extended near base of basiphallus, with basal stalk broad in lateral view. Hypandrium narrow.

Female. Unknown.

**Figure:**
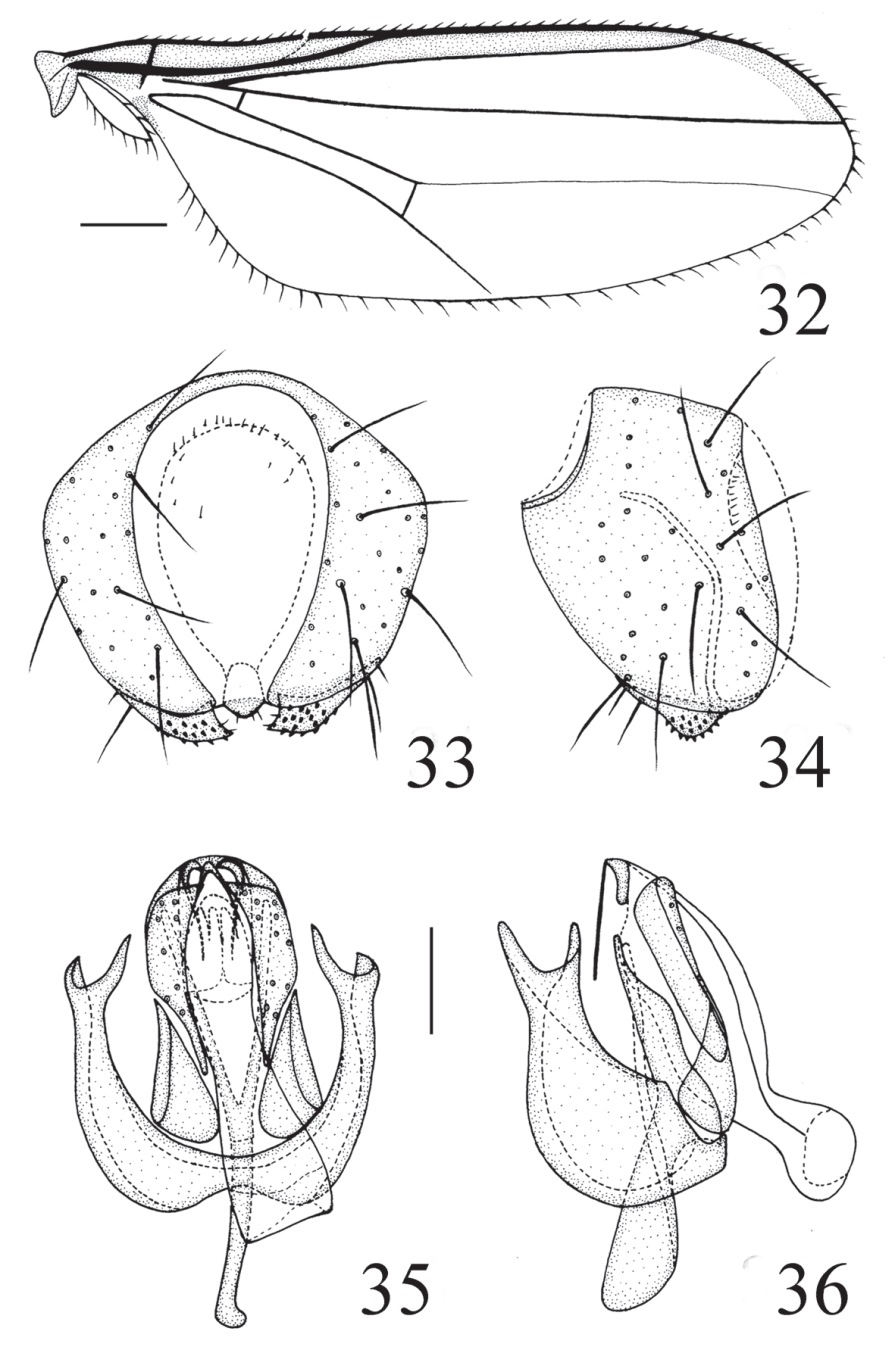
**Figures 32–36.** *Thressa longimaculata* sp. n., male. **32** wing **33** epandrium, posterior view **34** epandrium, lateral view **35** hypandrium and phallic complex, ventral view **36** hypandrium and phallic complex, lateral view. Scale bar = 0.2mm (32), Scale bar = 0.05mm (33–36).

#### Type material. 

Holotype ♂, Fujian: Wuyishan (26°54'N, 116°42'E, 850m), 17. VII. 2010, Xiaoyan Liu. Male type specimen was stored in 75% ethanol.

#### Distribution. 

China: Fujian.

#### Remarks. 

The new species is somewhat similar to *Thressa signifera* Walker in the wing with a brown spot near the wing apex, costal cell and marginal cell somewhat tinged with brown. But it can be separated from the latter by the katepisternum and katepimeron with the black lower portion; the paratergite and posterodorsal portion of the anepisternum with one triangular yellow spot; the femora entirely yellow. In *Thressa signifera*, the thoracic pleuron has a white band; the femora are black except yellow distally (Walker 1860, Nartshuk 1993).

#### Etymology. 

The specific name is from the Latin *longi-* (“long”) and *maculatus* (“maculate”), refers to the long brown spot near the wing tip.

### Thressa flavior

(Duda, 1934)

http://species-id.net/wiki/Thressa_flavior

Figs 37 44

Chalcidomyia flavior  Duda, 1934: 124. Type locality: Sumatra. (Holotypes deposited in Museum für Naturkunde, Berlin).
Thressa flavior  (Duda): Sabrosky 1977: 319; Nartshuk 1993: 114.

#### Diagnosis.

Scutum with a yellow transverse stripe anterior to scutellum, about 1/7 times as long as scutum. Paratergite and anepisternum with one oblique, wide, yellow stripe; dorsal portion of katepisternum with a small triangular yellow spot. Surstylus pipe-like. Cercus long, oblong in dorsal view.

#### Description.

Male.Body length3.0–3.5 mm, wing length2.4–2.9 mm.

Head (Figs 37, 38) black without microtomentum, about 0.8 times as long as high, wider than thorax; face sometimes concave in lateral view, bright brown with a yellow transverse stripe below antenna; epistoma yellow; frons black except for anterior 1/8 yellow, about 0.75 times as long as wide, projecting only slightly in front of eye, almost entirely occupied by broad ocellar triangle; gena black, narrow, about 1/10 times as high as postpedicel; vibrissal angle obtuse; parafacial black, linear; postgena black; clypeus light black. Ocellar triangle very large and broad, shiny black, smooth, reaching to anterior margin of frons with broad apex; ocellar tubercle black. Occiput black. Cephalic setae and setulae black, weakly developed; *if* extremely short, in 1 row on the surface of the triangle; *orb* very minute, upright; *oc* extremely small; *pvt* small hair-like, upright, convergent; *vte* shorter than *pvt* and *vti* indistinct. Antenna black with thick grayish microtomentum, but postpedicel with yellowish brown ventral surface; postpedicel 2 times as long as wide, parallel-sided; arista pectinate with 4 branches dorsally and 3 ventrally, apically with some short setulae, black except for basal segment yellow. Proboscis and palpus light blackish brown with blackish setulae.

Thorax (Figs 39, 40) black without microtomentum, with granulated microsculpture, evenly covered with short setulae. Scutum strongly convex, almost as long as wide, with a yellow transverse stripe anterior to scutellum, about 1/7 times as long as scutum. Thoracic pleuron black except for paratergite and anepisternum with one oblique, wide, yellow stripe; dorsal portion of katepisternum with a small triangular yellow spot. Scutellum about 0.7 times as long as wide; *ap sc* short, distinctly shorter than scutellum. Setae and setulae on thorax black. Legs yellow except for tarsomere 5 brown. Setulae on legs yellow, but apical portion of tarsi with some brown setulae. Tibial organ distinct, oblong. Wing about 2.7 times as long as wide, hyaline without brown spot near wing apex, costal cell somewhat tinged with brown; veins brown. Relative lengths of 2nd: 3rd: 4th costal sections = 5: 2: 1; discal cell narrow and long; crossveins r-m and m-m not approximate, r-m at basal 0.4 of discal cell. Halter pale yellow on knob, brown on stem.

**Figure:**
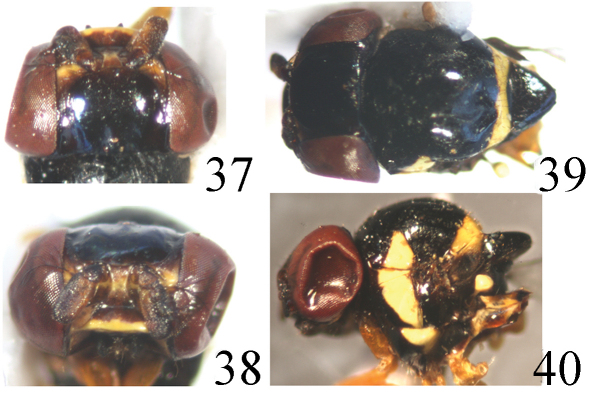
**Figures 37–40.** *Thressa flavior* (Duda), male. **37** head, dorsal view **38** head, facial view **39** mesonotum and scutellum, dorsal view **40** mesonotum and scutellum, lateral view.

Abdomen mainly yellow, tergite 2 posteriorly with two narrow black lateral stripes, basal 1/2 of tergite 3 with a transverse black stripe, rest of tergite 3 and following tergites with a mid-longitudinal black stripe; venter yellow. Setulae on abdomen black. Male genitalia (Figs 41–44): Epandrium short tubular, weakly sclerotized, yellow with long yellow setulae, with 2 band-like sclerites along posterodorsal notch; surstylus pipe-like, basally broad with short spinous setulae, distally narrowed, attaching to epandrium anteroventrally. Cercus long, oblong in ventral view. Gonites arranged in a row; postgonite long with some sensory setulae; pregonite slightly shorter than postgonite, basally broad, distally narrowed; basiphallus longer than wide, cylindrical; distiphallus cylindrical, longer than basiphallus, reaching to dorsal margin of hypandrium, largely membranous but weakly sclerotized on apical end; phallapodeme long, extended near base of basiphallus, with basal stalk broad in lateral view. Hypandrium narrow.

Female. Unknown.

**Figure:**
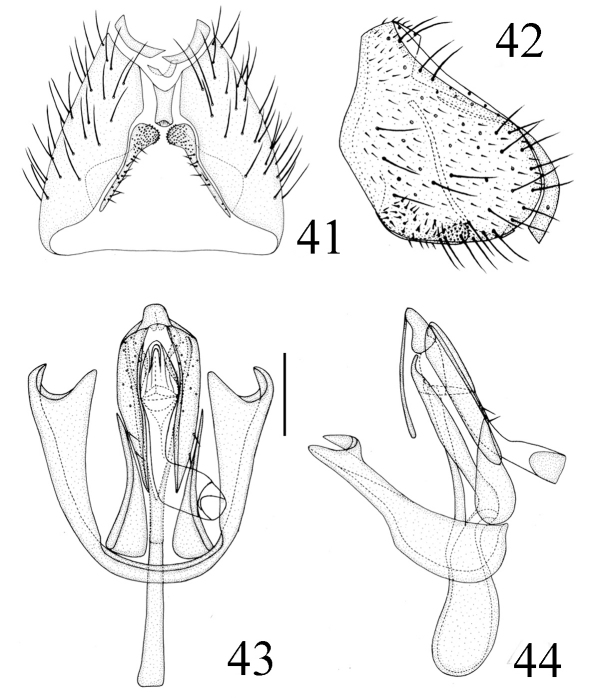
**Figures 41–44.** *Thressa flavior* (Duda), male. **41** epandrium, ventral view **42** epandrium, lateral view **43 **hypandrium and phallic complex, ventral view **44** hypandrium and phallic complex, lateral view. Scale bar = 0.1mm.

#### Specimens examined.

Holotype ♂, Sumatra, 1924, E. Jacobson; 1 ♂, Yunnan: Menglun (21°55'N, 101°13'E, 630m), 10. III. 1999, Ding Yang; 1 ♂, Hainan: Wuzhishan (18°50'N, 109°42'E, 600m), 16. V. 2007, Kuiyan Zhang. Type specimen pinned thoracic pleuron, other specimens dry mounted on label laterally on right thorax.

#### Distribution. 

China: Yunnan, Hainan; Indonesia; Malaysia.

#### Remarks. 

The species is somewhat similar to *Thressa polita* (de Meijere) in the scutum with a yellow transverse stripe anterior to the scutellum. But it can be separated from the latter by the abdomen being mainly yellow, tergite 2 posteriorly with two narrow black lateral stripes, basal 1/2 of tergite 3 with a transverse black stripe, rest of tergite 3 and following tergites with a mid-longitudinal black stripe; the legs entirely yellow. In *Thressa polita*, the abdomen is mostly black except for the basal portion and posterior margin yellow; the legs are yellow except the femora partly black (de Meijere 1910; Nartshuk 1993 ).

### Thressa beckeri

(de Meijere, 1913)

http://species-id.net/wiki/Thressa_beckeri

Hemisphaerisoma politum  Becker, 1911: 47. Type locality: Taiwan (Syntypes deposited in Hungarian Natural History Museum, Budapest)
Chalcidomyia beckeri  de Meijere, 1913: 292.
Thressa beckeri  (de Meijere): Sabrosky 1977: 319; Spencer 1986: 609; Nartshuk 1993: 117.

#### Diagnosis.

 Postpedicel narrow and elongate, 3 times longer than wide.Legs yellow, but entire femora yellow in male; black centrally in female. Wing hyaline with a brown spot near wing apex. Abdomen entirely dark, bluish black.

#### Distribution.

 China: Taiwan; Indonesia; Philippines; Australia.

#### Remarks.

 Becker (1911) firstly described *Hemisphaerisoma politum* from Taiwan and gave the figure of the head. De Meijere (1913) transferred it to *Chalcidomyia*, and gave the new name, *Chalcidomyia beckeri* (*Chalcidomyia politum* preoccupied by *Chalcidomyia polita* de Meijere (1910)). *Thressa beckeri* (de Meijere) was treated as synonymy with *Thressa punctifera* (de Meijere) from Java by Duda (1934), but Sabrosky (1977) accepted them as two distinct species.

### Thressa cyanescens (Becker, 1916)

http://species-id.net/wiki/Thressa_cyanescens

Chalcidomyia cyanescens  Becker, 1916: 440. Type locality: Taiwan (Holotypes deposited in Hungarian Natural History Museum, Budapest).
Thressa cyanescens  (Becker): Sabrosky 1977: 319; Kanmiya 1983: 236; Nartshuk 1993: 114.

#### Diagnosis.

Thorax entirely shiny black. Legs yellow except fore coxae black, in male fore femora black except distal part yellow; in female, all femora black except distal part yellow. Wing hyaline without a brown spot near wing apex. Epandrium with 2 narrow bands of sclerite along posterodorsal notch. Cercus small with a concavity on ventral margin.

#### Distribution.

 China: Taiwan; Japan.

#### Remarks.

 Becker (1916) firstly described *Chalcidomyia cyanescens* from Taiwan. Sabrosky (1977) transferred it to *Thressa*. Kanmiya (1983) newly recorded *Thressa cyanescens* from Japan, and gave the figures of male genitalia and abdomen lateral view.

### Thressa spuria

(Thomson, 1869)

http://species-id.net/wiki/Thressa_spuria

Geomyza spuria  Thomson, 1869: 599. Type locality: China (Holotypes deposited in Naturhistoriske Riksmuseet, Stockholm).
Thressa spuria  (Thomson): Sabrosky 1977: 319; Nartshuk 1993: 114.

#### Diagnose.

Frons slightly longer than wide (33:28). Femora black, tibiae and tarsi entirely yellow. Wing hyaline without a brown spot near wing apex. Surstylus short, nearly triangular. Pregonite as long as postgonite.

#### Distribution. 

China; Pakistan.

#### Remarks.

 Thomson (1869) firstly described *Geomyza spuria* from China. Sabrosky (1977) transferred it to *Thressa*. Nartshuk (1993) gave the figures of male genitalia. I examined the photos of the holotype in the Naturhistoriske Riksmuseet.

### Thressa guizhouensis

Yang, 1992

http://species-id.net/wiki/Thressa_guizhouensis

Thressa guizhouensis  Yang, 1992: 315. Type locality: China (Syntypes deposited in Entomological museum of China Agricultural university).

#### Diagnosis.

Legs black except for distal part of femora, entire tibiae and tarsi yellow. Wing hyaline without a brown spot near wing apex. Abdomen shiny black. Surstylus short, triangular. Pregonite about 0.7 times as long as postgonite.

#### Specimens examined.

 Holotype ♂, Allotype ♀, Guizhou: Guiyang, 25. VII. 1987, Jikun Yang; 2 ♂♂, Fujian: Wuyishan, 2. VII. 2009, Li Shi; 6 ♀♀, 3 ♂♂, Fujian: Nanping, 18. VII. 2009, Xiaoyan Liu; 1♂, 1♀, Fujian: Wuyishan, 27. IX. 2009, Weina Cui; 2 ♂♂, Fujian: Wuyishan, 30. IX. 2009, Tingting Zhang; 3 ♂♂, 2 ♀♀, Fujian: Wuyishan, 18. VII. 2010, Xiaoyan Liu; 8 ♂♂, 3 ♀♀, Fujian: Wuyishan, 21–23. VII. 2010, Xiaoyan Liu.

#### Distribution.

 China: Guizhou, Fujian.

#### Remarks.

Yang (1992) firstly described *Thressa guizhouensis* from China and gave the figures of male genitalia.

### Thressa maculata

Yang, 1992

http://species-id.net/wiki/Thressa_maculata

Thressa maculata  Yang, 1992: 315. Type locality: China (Holotypes deposited in Entomological museum of China Agricultural university).

#### Diagnose.

 Leg yellow, except for fore and mid coxae black, fore femora blackish with yellow distally. Wing hyaline with a brown spot near wing apex. Abdomen shiny yellow except tergites 1–3 black. Surstylus short, incurved distally. Pregonite shorter, about 0.3 times as long as postgonite.

#### Specimens examined. 

Holotype ♂, Yunnan: Jinghong, 12. IV. 1981, Fasheng Li.

#### Distribution.

 China: Yunnan.

#### Remarks.

Yang (1992) firstly described *Thressa maculata* from China and gave the figures of male genitalia.

## Supplementary Material

XML Treatment for Thressa bimaculata

XML Treatment for Thressa daiyunshana

XML Treatment for Thressa foliacea

XML Treatment for Thressa longimaculata

XML Treatment for Thressa flavior

XML Treatment for Thressa beckeri

XML Treatment for Thressa cyanescens

XML Treatment for Thressa spuria

XML Treatment for Thressa guizhouensis

XML Treatment for Thressa maculata

## References

[B1] Andersson H (1977) Taxonomic and phylogenetic studies on Chloropidae (Diptera) with special reference to Old World genera. Entomologica Scandinavica,Supplement 8: 1-200

[B2] Becker Th (1911) Chloropidae. Eine monographische Studie. iii. Teil. Die indo-australische Region. Annales Historico-Naturales Musei Nationalis Hungarici 9: 35-170

[B3] Becker Th (1916) Neue Chloropiden aus dem Ungarischen National Museum. Annales Historico-Naturales Musei Nationalis Hungarici 14: 423-453

[B4] Becker Th de Meijere JCH (1913) Chloropiden aus Java. Tijdschrift voor Entomologie 56: 283-307

[B5] Duda O (1934) Fauna sumatrensis, Bijdrage No. 74, Chloropidae (Diptera). Tijdschrift voor Entomologie 77: 55-161

[B6] Kanmiya K (1983) A systematic study of the Japanese Chloropidae (Diptera). Memoirs of the Entomological Society of Washington 11: 1-370

[B7] De Meijere JCH (1910) Studien über südostasiatische Dipteren. IV, Die neue Dipterenfauna Von Krakatau. Tijdschrift voor Entomologie 53: 58-194

[B8] Nartshuk EP (1993) Chloropidae (Diptera) from Vietnam and South China. Trudy Zoologicheskogo Instituta 240: 77-120

[B9] Sabrosky CW (1977) Family Chloropidae. In: Delfinado MD Hardy DE (Eds). A catalog of the Diptera of the Oriental Region, 3.The University of Hawaii Press, Honolulu: 277-319 doi: 10.1080/00222938600770401

[B10] Spencer KA (1986) The Australian Chloropinae (Diptera: Chloropidae). Journal of Natural History 20 (3): 503-615

[B11] Thomson CG (1869) Diptera, Species novas descripsit. In: Kongliga svenska fregatten Eugenies resa omkring jorden, Stockholm, 443–614

[B12] Walker F (1860) Catalogue of the Dipterous insects collected at Makessar in Celebes by Mr A. R. Wallace, with descriptions of new species. Journal and Proceedings of the Linnean Society ( London) Zoology 4: 145-172

[B13] Yang D (1992) Two new species of *Thressa* from China (Diptera: Chloropidae). Acta Agriculturae Universitatis Pekinensis 18 (3): 315-316

